# Supplementary material for: Unmasking the impact: Racial microaggressions and the health consequences for Latinas in the United States
Source: PLOS Ment Health. 2025 Aug 26;2(8):e0000416. doi: 10.1371/journal.pmen.0000416 (PMC12798488; doi:10.1371/journal.pmen.0000416)
Supplement: Attachment — Submitted filename: Response to Reviewers_ PLOS_2025.pdf [file pmen.0000416.s002.pdf]

Dear Reviewers,

I would like to begin by expressing my gratitude for your thorough comments and suggestions regarding the recent (hopefully minor) edits. I have addressed all of your feedback, and our team is more than willing to make any additional changes or clarifications, as well as re-run any necessary statistical tables to reflect the dependent variables for each participant group. Please find our responses noted in blue ink.

We look forward to your feedback on this novel research.

Below you will find your comments and our responses and how they were addressed referencing the sentence line numbers in the tracked document. We hope this meets your expectations and we are open to addressing any further questions or comments as we move to publication.

Please include the following items when submitting your revised manuscript:

- A rebuttal letter that responds to each point raised by the editor and reviewer(s). You should upload this letter as a separate file labeled 'Response to Reviewers'. Uploaded to the portal ✓
- A marked-up copy of your manuscript that highlights changes made to the original version. You should upload this as a separate file labeled 'Revised Manuscript with Track Changes'. Uploaded to the portal✓
- An unmarked version of your revised paper without tracked changes. You should upload this as a separate file labeled 'Manuscript'. Uploaded to the portal ✓

Journal Requirements:

Please review your reference list to ensure that it is complete and correct. If you have cited papers that have been retracted, please include the rationale for doing so in the manuscript text, or remove these references and replace them with relevant current references. Any changes to the reference list should be mentioned in the rebuttal letter that accompanies your revised manuscript. If you need to cite a retracted article, indicate the article's retracted status in the References list and also include a citation and full reference for the retraction notice. This has been addressed at the end of this document.

Review Comments to the Author

Please use the space provided to explain your answers to the questions above. You may also include additional comments for the author, including concerns about dual publication, research ethics, or publication ethics. (Please upload your review as an attachment if it exceeds 20,000 characters) this is in relation to the reference list.

Abstract

• Move the background info in the Methods (1st three sentences) to the Objective section as this presents the Background or Significance for your study, then your current study Objective sentence will flow nicely. Completed ✓

• Focus on the US sample only (36 states/659 participants) and moving this detail from Methods to the Results. Done ✓

• Include DASS and PHQ in Methods (spell out instruments + acronyms) as a measure of mental health and physical health respectively, then sharing the findings as you currently have in the Results. Done✓

• Move your analytic method CCA to last sentence in Methods (currently in Results). completed ✓

• Move the specific findings on the RMAS subscales (1st two sentences) from Discussion to Results these are findings. Completed ✓

• Suggest adding a concluding sentence on implications of your findings for research or practice/policy – summarizing sentence from what you have in the main text. Added ✓

Introduction

• Suggest having your new section (The study of racial aggression has been growing since…") as a new paragraph – Done ✓

• In the Abstract you state that much of the research on microaggressions and health have been with Black populations and to a lesser extent Latinos and even lesser extend Latinas. Suggest describing this in your Intro to improve the transition between the paragraphs about what microaggressions are and their impacts on health and health disparities, and the paragraph on Latinos and Latinas. Added 99-102

• Suggest adding your study objective to the end of the Intro section – moving the research questions from the Methods section lines 170-175 to end the Introduction – completed ✓

Methods

• Include your study design - ✓line 140

• Suggest including more info about sampling from your response to reviewers, namely about the purposive sampling method that was used and that you included community, university and prolific samples (how are prolific different than community and university) – 147-51 Done ✓

• Put the acronyms for the scales when you first introduce them so you can use the acronyms going forward – Done ✓

• Line 126 add "RMAS" to 6 microaggression subscales. Thank you for providing the detail about these, suggest listing the subscales for DASS and PHQ as well. Added ✓ 175-189

• Please move Table 3 to the Methods section. You could also provide scoring in this table instead of text if space is an issue. Moved 276 ✓ I moved this table, which shows the results of the CCA for RMAS predicting Physical Health for function 1 of the CCA. It may appear unconventional to include the results table within the Methods section; however, we have relocated it here in accordance with your request.

• Suggest moving the scale scoring to earlier in the methods section where you describe each instrument, as well as whether higher scores denote better/worse health. Done ✓
• Suggest focusing only on the scales and sample you used for this study, no need to reference the full sample and other scales as they do not pertain to your research questions. Please specify which scales you are referring to, was it for the Cronbach Alpha?

• How long did it take to do the survey and did participants receive any incentives for participation? – Added ✓ 152-54
• What statistical software did you use for analysis? SPSS ✓ 174

Results
• This paper focuses on Latinas so would expect that 100% of the sample is female or that you included Latinas who do not identify as female (e.g. gender non-binary) in the study? Please clarify. Approximately 95% identified as female. We did include participants who did not identify as female as well. We had two-non binary, one non-binary AFAB (i.e., assigned female at birth, one agender, and one transgender male. 259-60✓
• Add in a new Table 1 with participant characteristics so the reader can get a fuller understanding of who participated – eg. You state that the sample was mainly students (34%) but we don't know what other categories were included. Clarified the other statistics that made up employment status and realized this initial statement was incorrect. We also added all participant characteristics Demographic Frequency table.✓ For the entire sample.

Dr. DeYoung, and I discussed the challenge since we ran various statistical analyses on this sample. We can use the RMAS tool to analyze the participants who responded to the RMAS questions alongside the DASS and PHQ measures. Since we performed two CCAs for this study, could you please specify which sample descriptives you would like us to provide results for if not the broader study descriptive statistics? We are happy to do so.

• 18-34 is a broad category please report any more specific age data if you have this available.✓ Added in the frequency table with all the HHI breakdown and described the two categories that made up this percentage, 283-84.
• Describe how representative your sample is compared to English speaking Latinas in the

US overall – you do this for income but not for age, marital status, education, etc. ✓ This was addressed in section 283-299 first paragraph in Results section.

• Suggest adding sub-headers for mental health and for physical health and moving the last two paragraphs of Results lines 328-338 to start off each section then go into detailed analysis – Completed ✓

Discussion

• It's not clear why LatCRT and RBF are starting out the Discussion section as theoretical frameworks typically are in the Introduction section to provide background/rationale for your study or Methods to provide justification for what variables/data you collected. As it reads now, these theories belong in Intro as RBF shows how racism can impact health. If you are including LatCRT and RBF to help explain or contextualize your findings, suggest editing the Discussion to link these theories to your findings. – Moved to the Intro ✓ 111-131

• Suggest adding the need to engage monolingual Spanish speaking Latinas in future research given your focus on microaggressions. Also given environmental microaggressions had a surprising finding, further research with Latinas from other environments (outside student status/universities) would be important to see whether this relationship persists – Added to line 453- 456✓

• The new text on 399 – 409 "The potential implications..." belongs in the later Public Health Implications section. Thank you for adding these practice implications – moved ✓

References

Retracted papers and changes to the reference list:

38. Remove not in final edits Gravlee, C. C. (2009). How race becomes biology: Embodiment of social inequality. *American Journal of Physical Anthropology*, *139*, 47-57. https://s3.amazonaws.com/academia.edu.documents/32409860/Gravlee_2009_American_Journal_of_Physical_Anthropology.pdf?AWSAccessKeyId=AKIAIWOWYYGZ2Y53UL3A&Expires=1549659667&Signature=BWzWVw0yBTJ4fB9ebEi4yqhcjqo%3D&response-content-disposition=inline%3B%20filename%3DHow_Race_Becomes_Biology_Embodiment_of_S.pdf

This article was retracted after reviewing the final edits, which revealed that it was not initially included in the body of the manuscript. Although this reference appeared in the reference list, it was not cited in the manuscript itself.

Three new references have been added to address the reviewers' questions regarding participant characteristics and to provide documentation that compares how our participants performed relative to the national population. This additional information will help us analyze how representative this group is as a sample.

41. Moslimani, M., Lopez, M. H., & Noe-Bustamante, L. 11 facts about Hispanic origin groups in the US. *Pew Research Center*. August 16, 2023.https://pewrsr.ch/44dSllZ

42. USA Facts teams. How has marriage in the US changed over time? Fewer Americans are marrying than ever before, and when they do, they marry later in life. February 11, 2025. https://usafacts.org/articles/state-relationships-marriages-and-living-alone-us/

43. Moslimani, M, & Mukherjee, S. Half of Latinas say Hispanic women's situation has improved in the past decade and expect more gains in *Pew Research Center*. May 15, 2024. https://www.pewresearch.org/race-and-ethnicity/2024/05/15/half-of-latinas-say-hispanic-womens-situation-has-improved-in-the-past-decade-and-expect-more-gains/
