# Supplementary material for: Unmasking the impact: Racial microaggressions and the health consequences for Latinas in the United States
Source: PLOS Ment Health. 2025 Aug 26;2(8):e0000416. doi: 10.1371/journal.pmen.0000416 (PMC12798488; doi:10.1371/journal.pmen.0000416)
Supplement: Attachment — Submitted filename: Response to Reviewers_7_2025.pdf [file pmen.0000416.s003.pdf]

Final revisions addressing reviewer #5’s comments (Author comments are in blue)

Particularly, the reviewer requests a fuller discussion of what might be considered a more counter-intuitive finding, and suggests a theoretical basis for it. The relevant section of their comments is reproduced below:

"Notwithstanding the merits of this manuscript, the following concerns have been noted for the attention of the authors: 1. This study **reflects a descriptive and analytical cross-sectional design,** and it is suggested that as part of the abstract and in particular the **methodology section** the design of the study should be stated to guide readers. – Added to line 37.

"2. It is noted further that as part of the findings of the study, the environmental sub-scale as part of the independent variables (RMAS), is found to act as a suppressor and protector on both the DASS and Physical Health Status thereby decreasing the overall effect of DASS and PHQ as evidenced in the results of the study. For instance, Latinas with a higher frequency of racial microaggression in these two forms had higher levels of depression and anxiety. However, when environmental microaggressions were present the predicted levels of depression and anxiety decreased. Suppressors act to help the model fit better, even if they have no direct relationship with the outcome. This finding is supported by theory, and it is suggested that this theory should be cited especially under the discussion section to back the claim.

"Environments are constantly changing, and as such, human beings have ample opportunity to adapt to different situations and self-regulate their behavior, cognitions, and affect in a way that is consistent with their goals. The area of emotion regulation is subsumed under the broader domain of self-regulation. Several reasons may account for these results. First, people who tend to reappraise situations may be more likely to appraise or reappraise their social interactions as positive and enjoyable, leading to more positive responses on measures of social well-being. In addition, reappraisal use is positively correlated with positive affect which in turn is associated with higher interpersonal functioning and greater social satisfaction. Lastly, when negative social interactions arise, the use of reappraisal may help reduce negative emotional reactivity, allowing for a more civil and less hostile interaction, in turn reducing the likelihood of negative social experiences. Taken together, the research suggests that reappraisal may be an adaptive and helpful strategy to use in social situations (Ríos-Rodríguez et al., 2024). This may have implications for rolling out interventions on coping strategies among these clinical populations."

Added more to the discussion lines 441-465.

Regarding the article you cited titled "Benefits for Emotional Regulation of Contact with Nature: A Systematic Review" (Ríos-Rodríguez et al., 2024), we appreciate your reference to the Emotional Regulation Model. However, we believe it does not align closely with the focus of our research. The authors utilized the Attention Restoration Theory (ART) and the Stress Reduction Theory (SRT), both of which are centered on the benefits of exposure to nature and their potential for alleviating stress. This perspective does not intersect with the issue of environmental racial microaggressions. Furthermore, the meta-analysis that examined 25 research articles studying self-regulation within the context of nature exposure in Europe, while valuable, does not appropriately fit our study's objectives or target population regarding self-regulation. We do believe, on the other hand, that the Rejection Identification Model (RIM), mentioned in our discussion, which encompasses an individual's ability to re-appraise their environment based on environmental microaggression cues, does play a role in this outcome.

We emphasized the Rejection Identification Model in our study to elucidate the seemingly unconventional outcomes, particularly showing environmental microaggressions functioning as a suppressor within our model. This approach closely aligns with research conducted among minoritized ethnic groups, which demonstrates that identifying with one's affinity group can foster resilience and emotional strength, particularly among Latinas in this case.

Moreover, concerning racial discrimination expressed through microaggressions, research suggests that effective coping mechanisms encompass social support from family, friends, and affinity groups, as well as developing an awareness of these microaggressions. Such sources of support and understanding can enhance one's comprehension of the challenges tied to systemic oppression and discrimination (Nadal, 2018; Manohar, 2015). In addition, Manohar, 2015 found that emotional support and social identity affirmation support in coping with everyday racial discrimination could be a helpful coping strategy.

Across message types, participants perceived high-quality messages as being more effective generally, more effective in achieving multiple goals, and in facilitating emotional improvement and collective esteem enhancement. Also, for person-centered messages, reappraisal and empathy mediated the influence of message quality on message effectiveness, multiple goal achievement, and affect change. While in the case of social identity affirmation messages, reattribution mediated the influence of message quality on message evaluation. Finally, source ethnicity and participants' reported number of inter-ethnic friends emerged as significant moderators (Manohar, 2015).

References added:

47

RíosRodríguez ML, Rosales C, Hernández B and Lorenzo M. Benefits for emotional regulation of contact with nature: a systematic review. 2024. *Front. Psychol.*15:1402885.doi: 10:3389/fpsyg.2024.1402885

48

Jaimi Garlington, Cass Shum, Gloria Wong-Padoongpatt, Laura Book; "What it do?" The effects of racial code-switching on industry turnover intention. *International Journal of Contemporary Hospitality Management.* 3 November 2023; 35 (11): 3864–3882. https://doi.org/10.1108/IJCHM-11-2022-1335

49

Nadal, KL. *Microaggressions and Traumatic Stress: Theory, Research, and Clinical Treatment*. American Psychological Association. Washington, DC. 2018.

50

Manohar, U. *Effective Support Provision for Coping with Everyday Racial Discrimination: An Assessment of Emotional Support and Social Identity Affirmation Support.* 2015. Ohio State University, Doctoral dissertation. *OhioLINK Electronic Theses and Dissertations Center*, http://rave.ohiolink.edu/etdc/view?acc_num=osu1440087611.
