# Supplementary material for: Unmasking the impact: Racial microaggressions and the health consequences for Latinas in the United States
Source: PLOS Ment Health. 2025 Aug 26;2(8):e0000416. doi: 10.1371/journal.pmen.0000416 (PMC12798488; doi:10.1371/journal.pmen.0000416)
Supplement: Attachment — Submitted filename: ResponseToReviewers_PMEN_D_24_00063.docx [file pmen.0000416.s001.docx]

Reviewer's Responses to Questions

**Comments to the Author**

1. Does this manuscript meet PLOS Mental Health’s [publication criteria](https://na01.safelinks.protection.outlook.com/?url=https%3A%2F%2Fc05y1x9s.r.us-east-2.awstrack.me%2FL0%2Fhttps%3A%252F%252Fjournals.plos.org%252Fmentalhealth%252Fs%252Fcriteria-for-publication%2F1%2F010f019145f5eb48-96d28639-85ef-48e9-8ea2-183766fd851f-000000%2FNShCYoiuA6falCUyMtFuf2SmVBY%3D171&data=05%7C02%7C%7Caac3384ed52a4b5098aa08dcbab2ce3e%7C84df9e7fe9f640afb435aaaaaaaaaaaa%7C1%7C0%7C638590524326440651%7CUnknown%7CTWFpbGZsb3d8eyJWIjoiMC4wLjAwMDAiLCJQIjoiV2luMzIiLCJBTiI6Ik1haWwiLCJXVCI6Mn0%3D%7C0%7C%7C%7C&sdata=yj5dkyq%2F2iTlR0qDf2BdtE%2BazQiHMAN1a2t1t07DxkE%3D&reserved=0)? Is the manuscript technically sound, and do the data support the conclusions? The manuscript must describe methodologically and ethically rigorous research with conclusions that are appropriately drawn based on the data presented.

Reviewer #1: Partly

Reviewer #2: Yes

Reviewer #3: Partly

2. Has the statistical analysis been performed appropriately and rigorously?

Reviewer #1: Yes

Reviewer #2: Yes

Reviewer #3: Yes

3. Have the authors made all data underlying the findings in their manuscript fully available (please refer to the Data Availability Statement at the start of the manuscript PDF file)?

The [PLOS Data policy](https://na01.safelinks.protection.outlook.com/?url=https%3A%2F%2Fc05y1x9s.r.us-east-2.awstrack.me%2FL0%2Fhttps%3A%252F%252Fjournals.plos.org%252Fmentalhealth%252Fs%252Fdata-availability%2F1%2F010f019145f5eb48-96d28639-85ef-48e9-8ea2-183766fd851f-000000%2FjM1CRfT6F4cwWTzlFf51h6atOmM%3D171&data=05%7C02%7C%7Caac3384ed52a4b5098aa08dcbab2ce3e%7C84df9e7fe9f640afb435aaaaaaaaaaaa%7C1%7C0%7C638590524326453677%7CUnknown%7CTWFpbGZsb3d8eyJWIjoiMC4wLjAwMDAiLCJQIjoiV2luMzIiLCJBTiI6Ik1haWwiLCJXVCI6Mn0%3D%7C0%7C%7C%7C&sdata=Z2chpGbBaCPuBE1SgrdM0wtLADjHgUTBBOhPXuRcOpo%3D&reserved=0) requires authors to make all data underlying the findings described in their manuscript fully available without restriction, with rare exception. The data should be provided as part of the manuscript or its supporting information, or deposited to a public repository. For example, in addition to summary statistics, the data points behind means, medians and variance measures should be available. If there are restrictions on publicly sharing data—e.g. participant privacy or use of data from a third party—those must be specified.

We added the raw data to a repository. Please specify what data points you would like me to add to the repository. I have all of it saved and can add whatever else is needed.

Reviewer #1: No

Reviewer #2: No

Reviewer #3: Yes

4. Is the manuscript presented in an intelligible fashion and written in standard English?

PLOS Mental Health does not copyedit accepted manuscripts, so the language in submitted articles must be clear, correct, and unambiguous. Any typographical or grammatical errors should be corrected at revision, so please note any specific errors here.

Reviewer #1: Yes

Reviewer #2: Yes

Reviewer #3: Yes

5. Review Comments to the Author

Please use the space provided to explain your answers to the questions above. You may also include additional comments for the author, including concerns about dual publication, research ethics, or publication ethics. (Please upload your review as an attachment if it exceeds 20,000 characters)

Note: changes referenced in this response are in the marked up version of the manuscript. Line numbers correspond to this version. Thank you.

Reviewer #1: I would like to extend my appreciation for the effort you have invested in this work.
- Expand the keywords. Please find expanded keywords on lines 55-57 to include 9 keywords
- Ensure proper in-text citation formatting for all references. Thank you for this flag, proper in-text citation using Vancouver formatting has been used and previous superscript notations have been changed.
- Clearly state the research aims, mentioning investigating the psychological dynamics of racism. The research aims have been stated on lines 170-175.
- In the introduction section, explain in detail about the research topic with reference to qualitative studies. Details have been added to lines 79-88
- Explain the method of determining the sample size. Added to lines 140-147, we used a prioripower analysis to determine the sample size needed to ensure adequate power.
- While you've mentioned the sample size and demographic characteristics, consider providing more details relevant to the research question. We added more details about the survey instruments used 120-139 and two more demographic details in 212-214
- Further clarification is needed to explain the sampling methods used. The principle investigator gathered information from Latinas for their perspectives on microaggressions as related to their mental and physical health and well-being. Participants were recruited through Prolific were targeted using purposive sampling. To capture Latinos and Latinas who did not fit the Prolific study criteria of having US residency and/or English fluency, purposive community sampling and snowballing non-probability sampling were used (Leedy & Ormrod, 2016). Using Qualtrics software three separate URLs were created to distinguish between the community, university participants, and Prolific samples. In addition, Qualtrics has a security feature that the primary researcher set up to protect this survey from being completed more than once by the same participant. For TWU students and those recruited via social media, participants’ inclusion criteria allowed male participants (Latinos) in addition to Latinas. Please advise if you would like this paragraph added to the manuscript.
- What statistical solutions have you used to control intervening variables and increase internal validity? We agree with Carlson and Wu (2012) about adopting a conservative approach to control variables. "Adding more CVs does not make a study more rigorous. Unless there is a very sound reason that including a specific CV accomplishes an unambiguous and meaningfully statistical control objective, studies adding CVs may confound, rather than enhance, the interpretations of findings. When in doubt, leave them out." (Carlson & Wu, 2012, pg.19) We decided not to control for any intervening variables because that was outside of the scope of this research study. This research question was investigating the possibility of a relationship between racial microaggressions and physical and mental health outcomes.
- Is your sample representative of the entire population? We decided to only study women, so it is not representative of the entire Latino population in the US. We also chose to conduct this study in English this time for various reasons but could add that to the discussion section. There is a need to offer such a study to the Spanish speaking population and also gather qualitative data to learn more about that population with regard to microaggressions
- Did you include population with a history of mental health issues? We did not, the sample was sent out to a representative group of the Latino population (Prolific support) and posted on three social media sites. The study itself did not mention mental health or microaggressions in the survey title so we would not create bias among Latinos who may have self-selected into our study.

That said Latinos face disproportionately high rates of mental health conditions such as anxiety and depression, and post-traumatic stress disorder, more than 1 in 5. And only 36.1% received care in 2021 compared to 52,15 of white patients ([Unidos.org](https://unidosus.org/blog/2024/03/23/rompiendo-barreras-dismantling-barriers-to-latino-mental-health-care/#:~:text=Latinos%20face%20high%20rates%20of%20mental%20health%20conditions,only%2036.1%25%20of%20Hispanics%20and%20Latinos%20received%20care.), March 23, 2024)
- With what rationale have you chosen your sample from the countries and states mentioned? We did not anticipate who would respond to the study or which states would have more participants interested in this study; these are the individuals who self-selected into the study. We decided as a group not to include those participants outside the US because it was outside the scope of this study and the literature review.
- Each section can start with a brief introduction summarizing the analysis performed and then present the key findings. Please advise as to which sections you are referring to? Do you mean each section of the manuscript or each analysis completed of which there are two? I believe they each have paragraphs discussing the key findings. Please provide more detail so we can provide the summaries you have requested.
- Explain about the limitations of online sampling in the discussion section. Addressed in 410-416.
- The explanation of the results can be richer. Hopefully it is richer now with additions made ot the discussion section page 19.
- It seems that you can use interdisciplinary perspectives (cultural sociology and cultural - psychiatry) to explain the results obtained. We added in more information about the rejection identification model which is a coping method in addition to Racial Battle fatigue (RBF) which is the result of constant discrimination throughout one’s life.
- Sample loss is one of the important limitations of your study. What statistical methods have you - used to prevent reducing the internal and external validity of the study? We added in Cronbach’s Alpha and the corresponding table 1 at the beginning
- You can mention the psychometric properties (e.g., Cronbach's Alpha) in a separate paragraph or table. See above
- Briefly mention the content of the informed consent form, highlighting aspects like the voluntary nature, anonymity, confidentiality, and right to withdraw. Mentioned lines 115-116
- In discussion briefly restate the research question and the theoretical framework used in the study. Added to lines 342-344
- delve deeper into the limitations of theories in differentiating between races. Added to discussion section 344-363.
- Emphasize the potential implications of the findings for racism prevention strategies. Added to discussion page 19

Reviewer #2: Thank you for the opportunity to peer-review “Unmasking the Impact: Racial Microaggressions and the Health Consequences for Latinas in the United States”. Herein, the authors describe the harm of microaggressions on racialized peoples in the U.S. Latinas within the U.S. completed three online surveys exploring microaggressions, mental well-being, and physical symptoms. A statistical analysis was completed which describes symptoms of anxiety and gastrointestinal issues as prevalent symptoms related to experiencing microaggressions. Significantly, the team identified that all symptoms were analyzed to be lessened in the context alongside environmental microaggressions.

This is a well-written piece, adding valuable evidence to a very timely and important discussion. The last paragraph on page 13 describes an example of what an environmental microaggression might constitute. It would be beneficial to consider adding a brief description or summary of what each of the 6 microaggression sub-scales entail early in the manuscript. Similar as to how the authors briefly described the SDoH on page 3. It will add context and help the manuscript stand on its own, as implications include educating communities on microaggressions. Thank you for this flag, it was added on page 5, lines 126-139.

Reviewer #3: Dear Authors, I congratulate you on coming up with a nice research topic & nice piece of work. Please find my comments below:

Methods: The aim & objectives are to be explicitly provided here (it's there in the abstract, though) – added to lines 170-173.

Results: There are some repetitions in the result section (repetition that sexualisation and low achievement have a positive influence on the model, while environmental factor has a negative influence on the overall model's effect size), which can be avoided- we deleted the repetition on lines 236-237. Thank you.

Discussion: The most unique finding of the paper is that the environment had the opposite effect, refining the relationship by acting as a suppressor. These findings need to be elaborated to highlight the context and perspective. I think talking about its items will also be useful for the readers. Regards, reviewer. Thank you for this feedback, we added more about this, please let us know if more is needed.

Thank you all for your time and valuable feedback, we hope this is up to your liking and look forward to getting this manuscript to where it needs to be published.

Kindly,

Jeannine Rios
